# Cementing technique for primary knee arthroplasty: a scoping review

**DOI:** 10.1080/17453674.2019.1657333

**Published:** 2019-08-27

**Authors:** Anders M Refsum, Uy V Nguyen, Jan-Erik Gjertsen, Birgitte Espehaug, Anne M Fenstad, Regina K Lein, Peter Ellison, Paul J Høl, Ove Furnes

**Affiliations:** a Department of Clinical Medicine, Faculty of Medicine, University of Bergen, Bergen;; b Department of Orthopaedic Surgery, Haukeland University Hospital, Bergen;; c Centre for Evidence-Based Practice, Faculty of Health and Social Sciences, Western Norway University of Applied Sciences, Bergen;; d Medical Library, University of Bergen, Bergen, Norway

## Abstract

Background and purpose — The optimal cementing technique for primary total knee arthroplasty (TKA) remains unclear. We therefore performed a scoping review based on available studies regarding cementation technique in primary TKA and unicondylar knee arthroplasty (UKA).

Patients and methods — A search in 3 databases identified 1,554 studies. The inclusion criteria were literature that studied cementing technique in primary TKA or UKA. This included cement application methods, full or surface cementing, applying cement to the bone and/or prosthesis, stabilization of the implant during curing phase, bone irrigation technique, drilling holes in the bone, use of suction, and the timing of cementation. 57 studies met the inclusion criteria.

Results — The evidence was unanimously in favor of pulsatile lavage irrigation, drying the bone, and drilling holes into the tibia during a TKA. All studies concerning suction recommended it during TKA cementation. 7 out of 11 studies favored the use of a cement gun and no studies showed that finger packing was statistically significantly better than using a cement gun. There is evidence that full cementation should be used if metal-backed tibial components are used. Applying the cement to both implant and bone seems to give better cement penetration.

Interpretation — There are still many knowledge gaps regarding cementing technique in primary TKA. There seems to be sufficient evidence to recommend pulsatile lavage irrigation of the bone, drilling multiple holes, and drying the bone before cementing and implant insertion, and applying cement to both implant and on the bone.

Aseptic loosening is the most common cause of revision after total knee arthroplasty (TKA) worldwide (Khan et al. 2016). Implant loosening appears to be a multifactorial event, but without preceding micromotion of the implant, aseptic loosening seems unlikely to occur (Goodman et al. 1994, Scuderi and Clarke 2005). Aseptic loosening may occur at the implant–cement interface (Kutzner et al. 2018), or at the bone–cement interface (Mann et al. 1997, Dahabreh et al. 2015).

Studies have shown that sufficient cement penetration and thickness is important to prevent implant micromotion (Miskovsky et al. 1992). Penetration of cement into the cancellous bone at 1.5 mm or less usually leads to higher radiolucency and lower tensile strength, which is associated with early implant micromotion (Walker et al. 1984, Mann et al. 1997, Waanders et al. 2010).

The cementing technique is multifactorial and includes: preparation of the bone before cementation; where, when, and how to apply the cement; and the curing and stabilization phase after installation (Endres and Wike 2011, Cawley et al. 2013). A study by Lutz and Halliday (2002) indicated a wide variation in cementing technique among orthopedic surgeons. This highlights the need for a general consensus based on evidence on how to cement a TKA, especially the tibial component, which has a 4 times higher risk of loosening than the femoral component in total knee arthroplasty (Furnes et al. 2007, Dyrhovden et al. 2017). We therefore performed a scoping review on available studies regarding cementing technique in primary TKA and UKA. Our aim was to investigate knowledge on cementing technique in primary knee arthroplasty and to identify eventual gaps in the knowledge that need more research.

## Method

For this study, we followed the recommendations from the Cochrane Collaboration (Higgins et al. 2011) and the Methodological Framework to approach a scoping review (Arksey and O’Malley 2005).

### Research question

From the aim we created 8 research questions:

1. What is the recommended cement application method?
2. Surface cementation or full cementation?
3. Should cement be applied to either bone or prosthesis or both?
4. What is the recommended irrigation method?
5. Is drilling holes into the tibial bone recommended?
6. Is peroperative suction recommended?
7. At which cement phase should cement be applied?
8. How should the implant be stabilized during the curing phase?

### Eligibility criteria

We included all literature from our search on cementing technique in primary TKA and UKA where the topic was consistent with the formulated research questions. All study designs were included except for case reports. Literature that studied the use of tourniquet, patellar component, and mixing method of the cement were excluded to sharpen the scope of the study.

### Information sources

The information search through the electronic databases OVID MEDLINE, OVID Embase, and Web of Science was last updated September 27, 2018 by 1 author (RKL). Subject headings for the specific database and free text terms were used with no restrictions to language, time, or format. The complete search strategies are shown in Appendix 1, see Supplementary data. Keywords and free text terms were decided and validated by 3 of the authors (UN, AR, and OF).

### Study selection

The references were deduplicated in Endnote, and in addition manually by 2 of the authors (UN, AR). Obviously irrelevant studies were identified and excluded through title and abstract screening. 2 reviewers (UN, AR) independently screened the remaining studies and checked the full text versions of potential relevant studies.

### Data collection

The reviewers developed a data extraction sheet based on the Cochrane Consumer and Communication review group’s data extraction template (Ryan et al. 2015) and pilot tested it on 3 studies regarding use of drilling holes.

The result was discussed with the third reviewer (OF) for optimization and to decide which variables needed to be extracted from the studies.

### Data items

The parameters the reviewers (UN, AR, OF) agreed upon initially formed the aim of the study: study method, study design, demographics, follow-up period, level of evidence based after Oxford Centre for Evidence-based Medicine—Levels of Evidence (Howick et al. 2016), application method, preparation of the bone, cement type, prosthesis design, and outcome. Together, the 2 reviewers determined the studies’ Level of Evidence. The references were rated from I to V based on their study method. Animal and laboratory studies were regarded as mechanism-based reasoning or bench research and, therefore, graded as Level V (Howick et al. 2016).

Any disagreements were resolved by consensus or through the third reviewer.

### Funding and potential conflicts of interest

No funding was received. The authors declare no conflicts of interest.

## Results

### Study selection, quality, and study characteristics

**Figure 1.**
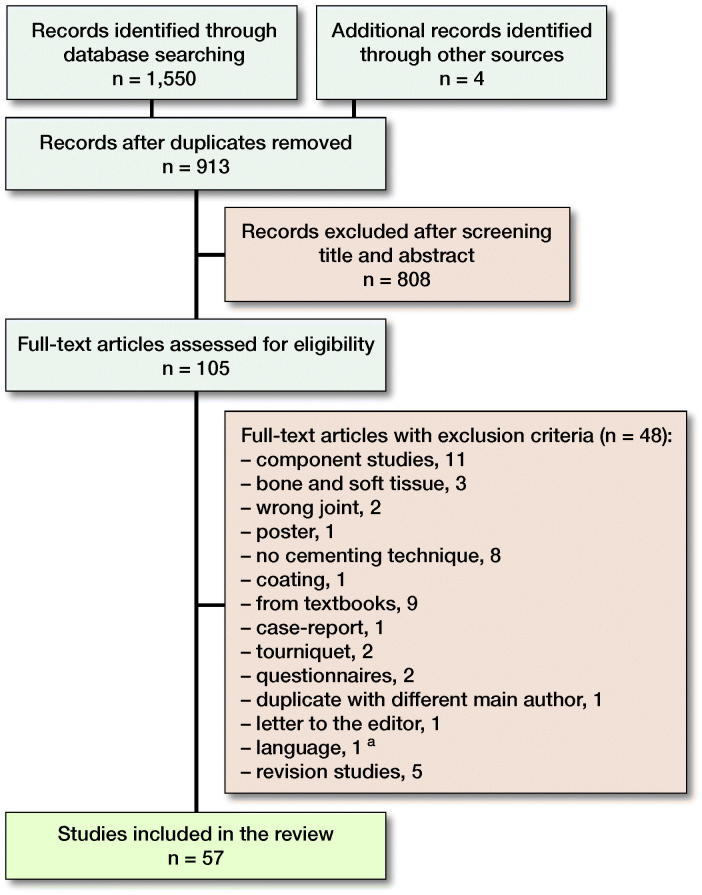
Flow diagram of study inclusion. **^a^** Pujol et al. (2008).

**Figure 2.**
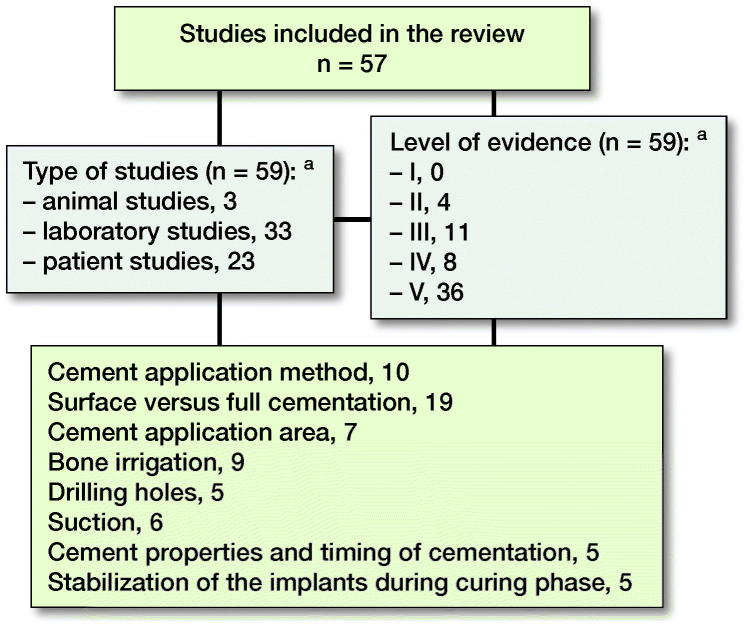
Included articles, study design, study quality, and inclusion groups. Some studies analyzed more than one parameter and were therefore categorized into several groups. **^a^** Walker et al. 1984 and Kanekasu et al. 1997 consists of 2 studies.

Of 1,554 studies 105 articles were retrieved in full text (Figure 1). 57 articles met the inclusion criteria: animal studies (n = 3), laboratory studies (n = 33), and clinical trials (n = 23). 2 studies had methods that met the inclusion criteria in 2 categories (Walker et al. 1984, Kanekasu et al. 1997). Only 4 studies were randomized controlled trials (RCTs). Characteristics of the included studies are summarized in Figure 2.

4,120 knees were included in the studies: 3,418 of them in patients, 501 in cadaver bones, and 111 sawbone knees. 25 of the knees were made from an experimental model (Bert and McShane 1998, Bucher et al. 2015). One study constructed a computer model of a female knee (Chong et al. 2011) and one study used finite element analysis (Cawley et al. 2012).

### Chapter 1. Cement application method

#### Studies

10 studies conducted between 2003 and 2017 were reviewed (Table 1, see Supplementary data). The studies consisted of 3 clinical, 4 sawbone, and 3 cadaver studies. Mostly, the aim was to compare different application methods; cement gun, spatula, and finger packing or syringe use to achieve optimal cement penetration.

6 studies favored the use of a cement gun (Labutti et al. 2003, Kopec et al. 2009, Lutz et al. 2009, Vanlommel et al. 2011, Bucher et al. 2015, Schlegel et al. 2015a). 4 of these studies favored use of cement gun over finger packing when comparing cement penetration, clinical function score after operation, mechanical pull-out force, and the occurrence of postoperative radiolucent lines (RLL) (Kopec et al. 2009, Lutz et al. 2009, Vanlommel et al. 2011, Schlegel et al. 2015a).

4 studies favored the use of finger packing (Perez Mananes et al. 2012, Schlegel et al. 2014, Silverman et al. 2014, Han and Lee 2017). 2 of these studies favored finger packing over use of a cement gun when comparing cement penetration on human cadaver tibia (Schlegel et al. 2014, Silverman et al. 2014). Schlegel et al.(2014) also studied lift-off force. No studies favored the use of spatula over any other methods in terms of cement penetration. 1 study favored use of syringe over finger packing comparing cement penetration and RLL (Lutz et al. 2009).

#### Comments

In the 2 studies where finger packing was recommended over the usage of a cement gun (Schlegel et al. 2014, Silverman et al. 2014), the finger packing method was accompanied by factors that might be considered favorable, such as pulsatile lavage preparation of the tibial bone and cementing in doughy phase. The studies favoring finger packing still showed acceptable results, but it seems that use of a cement gun has shown better results in terms of surrogate outcomes. None of the studies could show any reduction in loosening rate when using a cement gun. In terms of the optimal cement penetration, Vanlommel et al. (2011) suggest a penetration between 3 and 5mm. Walker et al. (1984) concluded in their study that cement penetration over 1.5 mm is sufficient but suggested that ideally the penetration should be between 3 and 4 mm. None of the studies showed an increased loosening or revision rate with lower cement penetration, but Miller et al. (2014) concluded that a cement mantle over 3 mm is advisable to counteract cement decay over time. A pragmatic view would be to aim for between 3 and 5 mm cement penetration.

### Chapter 2. Surface versus full cementation

#### Studies

19 studies were reviewed (Table 2, see Supplementary data). These studies consisted of 11 clinical studies (3 of them RCTs), 2 sawbone studies, 5 cadaver studies, and 1 computer study. The aims of these studies were either to compare the impact of full or surface cementation or to assess the quality of one of these methods.

2 studies showed a statistically significant difference when comparing surface cementation (SC) against full cementation (FC) favoring FC in terms of lift-off and rotation when using a metal-backed tibial model (Hyldahl 2003) and lift-off when using mobile-bearing prosthesis (Luring et al. 2006). 8 clinical and laboratory studies reported no statistically significant difference when comparing the 2 techniques. 1 study showed lower lift-off force using SC if the cement mantle was less than 3 mm, but no difference if the mantle was above that depth (Bert and McShane 1998). Other studies showed that FC gave a higher tibial bone resorption (Chong et al. 2011) and more micromotion (Skwara et al. 2009, Cawley et al. 2012). 2 studies showed that FC gave a higher stability and less strain compared to SC, especially in mobile bearing TKAs (Luring et al. 2006, Cawley et al. 2012). Finally, 1 study showed an excellent 10-year clinical result for both SC and FC, but found a lower revision rate for mechanical reasons in SC (Galasso et al. 2013), whereas Schlegel et al. (2015b) found no such difference. Case-control studies showed that both techniques could be sufficient over time, but without randomization, large number of patients, and longer follow-up this information was hard to assess (Galasso et al. 2013). However, an RCT using radiostereometric analysis (RSA) by Hyldahl et al. (2003, 2005a, 2005b) compared FC with SC in metal-backed and all-polyethylene tibial components of the AGC knee. The studies found that migration was reduced when using FC in metal-backed tibial components, but the migration was the same for all-polyethylene tibial components. The result in metal-backed components could not be confirmed by Saari et al. (2009) using the Profix metal-backed knee replacement.

#### Comments

The question of full versus surface cementation seems to be the most controversial and more clinical studies are needed. 3 studies showed that FC was better than SC (Bert and McShane 1998, Hyldahl 2003, Luring et al. 2006); meanwhile most of the other studies had the 2 techniques as equal.

The different prosthesis designs, such as coating, roughness of the prosthesis surface, metal type, metal or all-poly tibial components, use of mobile bearing, and keel type probably influenced the results in the comparison of full versus surface cementing (Hyldahl 2003, Hyldahl et al. 2005a, 2005b, Luring et al. 2006, Saari et al. 2009). More clinical studies comparing both techniques in a standardized study method with different implants would be advisable to make progress on this topic.

### Chapter 3. Cement application area

#### Studies

7 studies were reviewed (Table 3, see Supplementary data). These studies consisted of 2 clinical studies, 2 sawbone studies, 2 cadaver studies, and 1 porcine study. The aims of these studies were to assess the cement–bone interface strength, cement penetration depth and cement–mantle thickness regarding application of the cement to the bone only, implant only, or both.

4 studies favored application onto both the bone and prosthesis over application onto only either bone or prosthesis alone, where cement penetration and the length of the cement mantle was compared (Stannage et al. 2003, Vaninbroukx et al. 2009, Vanlommel et al. 2011, Wetzels et al. 2018). 1 study found no statistically significant difference comparing application onto bone versus bone and prosthesis, when studying properties of the cement interface and mechanical load to failure using a UKA model (Grupp et al. 2013). Another study favored cement application onto the prosthesis only over cement application onto the bone only, comparing percentage of cement penetration at different levels in porcine tibial bone (Bauze et al. 2004). Regarding the femoral component, 2 studies reported that cementation onto both the bone and the prosthesis was superior to cement application only to the bone or prosthesis. However, in only 1 of them was the result statistically significant (Vaninbroukx et al. 2009).

#### Comments

At this point, a technique applying the cement to both implant and bone seems to be more favorable as supported by Vaninbroukx et al. (2009), Vanlommel et al. (2011), Han and Lee (2017), and Wetzels et al. (2018). More studies analyzing only this parameter are needed. These studies should also include the timing of application of cement to the implants and bone.

### Chapter 4. Bone irrigation

#### Studies

9 studies were reviewed (Table 4, see Supplementary data). These studies consisted of 2 clinical studies and 7 cadaver studies. The aim of these studies was to compare different methods of preparing the bone before cementation. These methods were mainly irrigation with syringe, brush, lavage, or no preparation. 8 studies favored pulsatile lavage over manual syringe. Cement penetration depth, bone–cement interface strength, and pull-out force were statistically significantly increased when the bone was pulsatile lavaged compared with brushed or syringe lavaged (Ritter et al. 1994, Maistrelli et al. 1995, Clarius et al. 2009, Schlegel et al. 2011, Jaeger et al. 2012, Helwig et al. 2013, Schlegel et al. 2014, Boontanapibul et al. 2016, Scheele et al. 2017). One study found no difference between pulsatile lavage and cleaning with a surgical brush comparing cement penetration and a mechanical compression test using a UKA model (Scheele et al. 2017). 1 study found cleaning with pressurized CO2 in addition to pulsatile lavage to be significantly better than pulsatile lavage alone (Boontanapibul et al. 2016).

#### Comments

All 9 studies on irrigation methods of the bone concluded that pulsatile lavage was superior to irrigation by syringe. To achieve sufficient cement penetration depth and to reduce the occurrence of RLLs, a clean bone by pulsatile lavage and drying afterwards is crucial for the initial stability of the components (Schlegel et al. 2011). All included studies showed an improvement in either cement penetration or reduction in RLL. None of the studies showed reduction in the primary outcome loosening or revision rate. Our review showed that TKA studies regarding bone irrigation were unanimously in favor of pulsatile lavage irrigation, which therefore should be performed routinely in TKAs.

### Chapter 5. Drilling holes

#### Studies

5 studies were reviewed (Table 5, see Supplementary data). These studies consisted of 2 clinical studies, 2 cadaver studies, and 1 dog study. In 2 studies, drilling holes were compared with no drilling holes (Miskovsky et al. 1992, van de Groes et al. 2013). The diameter of the drilling holes ranged from 2.4 to 4.5 mm. The numbers of holes were stated in 3 studies and the depth was mentioned in 4 out of 5 studies. All studies favored drilling holes into the tibial bone as this increased cement penetration, reduced occurrence of RLL, and increased bone–cement interface strength. No clinical studies examined or showed reduced loosening rate. None of the included studies discarded the measure of drilling holes into the bone due to negative effects. Only 1 of the studies compared different diameter of drilling holes and concluded that 4.5 mm diameter holes were superior to 2.0 mm holes in a sclerotic medial tibial plateau (Ahn et al. 2015).

#### Comments

The optimal number of holes, depth, and size should be further investigated and their clinical effect on loosening rate should be verified.

### Chapter 6. Suction

#### Studies

6 studies were reviewed (Table 6, see Supplementary data). These studies consisted of 3 clinical studies, 1 sawbone study, and 2 cadaver studies. The aim of these studies was to assess the effect of applying negative pressure to the tibial bone on cement penetration.

The study by Banwart et al. (2000) compared negative pressure intrusion (NPI) against standard third-generation positive pressure intrusion (PPI) with no difference in cement penetration. The NPI technique was described similarly as a suction technique via Wolf needle and PPI was described as a standard third-generation cementing technique with a cement gun.

All studies recommended using NPI but only 3 studies showed statistically significantly higher cement penetration using suction compared with no use of suction (Norton and Ayres 2000, Stannage et al. 2003, Bucher et al. 2015). No studies of suction has shown reduced loosening.

#### Comments

The use of suction in the tibia probably cannot replace a cement gun, but it might be a viable addition to optimize cement penetration depth if a tourniquet is not used. In this study suction and NPI were regarded as the same technique.

### Chapter 7. Cement properties and timing of cementation

#### Studies

5 studies were reviewed (Table 7, see Supplementary data). These studies consisted of 1 clinical study, 3 cadaver studies, and 1 study that involved both a cadaver and a radiographic study (Walker et al. 1984). The aim of these studies was mainly to compare different cement application timings or cement phases and what effect these methods had on the cement–bone interface, RLL, and cement penetration. 1 study recommended that the cement should be applied in a doughy phase, comparing cement penetration and the use of a cement gun and finger packing (Silverman et al. 2014). 1 study highlighted the importance of application time when creating a cement–cement interface comparing mechanical bond strength and scanning electron microscope analysis (Park et al. 2001). 1 study concluded that a cement mantle over 3 mm is advisable to counteract decay over time comparing cement depth and contact fraction in post mortem TKAs (Miller et al. 2014). Dahabreh and colleagues’ study (2015) highlighted the diversity between cement brands and the study by Walker et al. analyzed many aspects to find the ideal cement penetration.

#### Comments

It is important to use the manufacturers’ advice on cement curing, since different cement types have different properties (Kühn 2000, Dahabreh et al. 2015). In summary, to generate a strong bone–cement and cement–cement interlock the application should take place at around 2–3 minutes in a doughy/application phase and the cement mantle should be at least 3 mm to weigh against the decay in the interlock over time (Miller et al. 2014). Park et al. (2001) show that creating a cement–cement interface was only 8% weaker than bulk cementation when created after 1 minute, whereas when created after 6 minutes was 42% weaker with only 50% bonding according to SEM analysis. After our literature search, Billi et al. (2019) published a laboratory study that recommended cementation of both the keel and undersurface of the tibial component, studying Palacos and Simplex cement. They also found that timing of cementation was important with improved pull-out force needed to separete the implant from the cement when the cement was applied on the implant in a sticky face 2 minutes after the start of mixing the Palacos cement and 3 minutes for the Simplex cement. The study also revealed that cementation in a dry condition gave higher pull-out force.

### Chapter 8. Stabilization of the implants during curing phase

#### Studies

5 studies were reviewed (Table 8, see Supplementary data). These studies consisted of 3 clinical studies, where 1 was an RCT, 2 were cadaver studies, and 1 was a porcine study. 1 study consisted of both a clinical and a cadaver study (Kanekasu et al. 1997). The aim of these studies was mainly to study different ways of keeping the prosthesis in position during the curing phase.

3 of the studies recommended using an external pressurizer to stabilize the implants during curing phase when compared against a manual method in a 2-stage cementation technique to increase cement penetration and stiffness (Kanekasu et al. 1997, Bauze et al. 2004, Diaz-Borjon et al. 2004). However, only 1 of these studies reported a statistically significant difference when using an experimental clamp, in the form of uniform stiffness of the fixation (Bauze et al. 2004). 1 study reported that with a single-stage cementation technique of UKA, a flexion angle of the knee of more than 45 degrees led to a tilting of the tibial component comparing femoral force application and cement penetration pressure (Jaeger et al. 2012). Single-stage cementing technique was superior to 2-stage cementing technique in 1 study, reducing the total number of RLLs (Guha et al. 2008).

#### Comments

Most surgeons do a single-stage cementing technique and extend the knee fully to apply pressure during the cement curing as described by Guha et al. (2008). But more evidence is needed to support this and also in which position the leg should be held when stabilizing the implant during the curing phase.

## Discussion

One of the most important findings in this scoping review was the heterogeneity between the studies. Comparability was limited due to different methods, materials, components, and parameters studied. 34 of the 57 included studies were laboratory studies and animal studies. The overall level of evidence seems low considering the potential impact on outcome. The most obvious gap in the literature is the lack of randomized clinical trials. We found only 4 RCTs and a lack of studies with revision or loosening as primary outcome. More research and especially solid RCTs are needed before one can find best practice.

### Summary

Based on our scoping review the following guidelines for the cementing technique can be recommended:

1. A cement gun can be recommended to achieve optimal cement penetration and reduce occurrence of RLLs. The optimal cement penetration is not clearly defined but studies indicate between 3 and 5 mm. Applying cement by finger packing is a satisfactory method, while applying cement with a spatula was not advisable.
2. Full cementation should be applied on both the stem/keel and undersurface of the tibial component if using metal-backed components. All-poly tibial components can be cemented with surface cementation.
3. Cement should be applied to both implant and bone. Applying cement on only the bone or prosthesis should be avoided.
4. Pulsatile lavage irrigation and drying of the bone should be performed routinely in TKA to increase cement penetration depth and bone–interface strength.
5. Drilling holes into the sclerotic bone surface of the tibia can be recommended.
6. Suction in the tibial bone shows promising results in terms of cement penetration, but the evidence is insufficient to recommend use of suction routinely in TKA.
7. The cement should be applied in the cement’s application phase to both the femoral and tibial bone.
8. A single-stage cementation procedure is the recommended technique with the knee extended, keeping it as immobilized as possible. There is uncertainty on the degree of extension needed.
9. There is evidence from in vitro studies that applying the cement to the implant early, 2 minutes after mixing, increases the implant cement bonding, but no clinical studies support this.

### Supplementary data

The Appendix and Tables 1–8 are available as supplementary data in the online version of this article, http://dx.doi.org/­10.1080/17453674.2019.1657333

## Supplementary Material

Supplemental Material
