# Supplementary material for: Cementing technique for primary knee arthroplasty: a scoping review
Source: Acta Orthop. 2019 Aug 27;90(6):582–9. doi: 10.1080/17453674.2019.1657333 (PMC6844414; doi:10.1080/17453674.2019.1657333)
Supplement: Supplemental Material [file IORT_A_1657333_SM8084.pdf]

Supplementary data

Appendix 1

Database: Ovid MEDLINE(R) and Epub Ahead of Print, In-Process & Other Non-Indexed Citations and Daily < 1946 to September 25, 2018 > September 27, 2018

----

1 Arthroplasty, Replacement, Knee/ (20013)
2 (knee prosthes* or knee replacement* or knee arthroplast* or TKR or TKA or UKR or UKA).ti,ab,kw. (28080)
3 1 or 2 (31066)
4 Bone Cements/ (10697)
5 Cementation/ (5140)
6 (cement* adj2 (technique* or method* or applic* or apply* or administrat*)).ti,ab,kw. (1658)
7 (technique* or method* or applic* or apply* or administrat*).ti,ab,kw. (7624658)
8 4 or 5 (15186)
9 7 and 8 (7483)
10 6 or 9 (8227)
11 3 and 10 (564)

Database: Embase < 1974 to 2018 September 26 > September 27, 2018

----

1 exp knee arthroplasty/ (21860)
2 (knee prosthes* or knee replacement* or knee arthroplast* or TKR or TKA or UKR or UKA).ti,ab,kw. (35316)
3 1 or 2 (40714)
4 exp bone cement/ (13684)
5 cementation/ (3763)
6 4 or 5 (17205)
7 (technique* or method* or applic* or apply* or administrat*).ti,ab,kw. (10279195)
8 6 and 7 (8880)
9 (cement* adj2 (technique* or method* or applic* or apply* or administrat*)).ti,ab,kw. (2264)
10 8 or 9 (10038)
11 3 and 10 (725)

Web of Science (Clarivate) Indexes = SCI-EXPANDED, SSCI, A&HCI, ESCI Timespan = All years 27. sept. 2018

----

1 – 33,326 – TOPIC: (“knee prosthes*” or “knee replacement*” or “knee arthroplast*” or TKR or TKA or UKR or UKA)
2 – 7,592 – TOPIC: (cement* NEAR/5 (technique* OR method* OR applic* OR apply* OR administrat*))
3 – 261 - #2 AND #1

Table 1. Cement application method

Description	LoE	Material	Method of study	Outcome	Conclusion
Bucher et al. (2015)	V	20 sawbones, experimental model<br><br>Group 1: 5<br>Group 2: 5<br>Group 3: 5<br>Group 4: 5	Group 1: Tourniquet and application with spatula<br>Group 2: No tourniquet and application with spatula<br>Group 3: No tourniquet and cancellous suction and application with spatula<br>Group 4: No tourniquet, cancellous suction and application with cement gun	Cement penetration (SD) in cm^2 centrally and peripherally, respectively:<br>Group 1: 3.2 (0.4) and 2.1 (0.2)<br>Group 2: 2.7 (0.4) and 1.9 (0.2)<br>Group 3: 3.4 (0.3) and 2.4 (0.3)<br>Group 4: 4.3 (0.4) and 3.2 (0.2)<br>Group 2 had significantly lower cement penetration than the rest, both centrally and peripherally (p < 0.0001–0.008).<br>Group 4 had significantly higher central and peripheral cement penetration compared with the rest (p < 0.0001).	The use of cement gun, no tourniquet and suction give good penetration during TKA compared with the use of spatula.
Han and Lee (2017)	III	734 TKAs [a] in 486 patients<br><br>Group 1: 403<br>Group 2: 331	Group 1: Cement applied on distal, anterior cut surface and posterior part of femoral component without digital pressurization<br>Group 2: Cement applied on distal and anterior cut surface with finger packing and whole cement surface of the femoral component	Group 2 had less radiographic loosening (p = 0.02), less revision for aseptic loosening (p = 0.03) and a significant difference in survival when radiological femoral component loosening was set as endpoint in univariable analysis, HR 4.2, (1.3–14.2) (p = 0.02) and multivariable analysis, HR 3.1 (1.0–12.2) (p = 0.048).	Using finger packing and full cementation of the femoral component and bone gives less loosening.
Kopec et al. (2009)	III	82 TKAs in 77 patients<br><br>Group 1: 41<br>Group 2: 41	Group 1: Application with cement gun<br>Group 2: Application with finger packing	Radiographic image analysis at 6 weeks: Group 1 had a significant higher cement penetration 1 of 7 zones (p = 0.002).<br>No statistical significant difference in KSFS [b], KSAS [c] and ROM [d] after 1 year.	Using a cement gun showed a higher cement penetration in 1 of 7 zones. No difference in RLLs [e] or function.
Labutti et al. (2003)	V	12 sawbones<br><br>Group 1: 6<br>Group 2: 6	Group 1: Application with a 65° angled pressure injector attached to cement gun<br>Group 2: Application with a normal cement gun	Higher mean penetration in 8 of 8 zones for Group 1. Only significant in 2 of the zones (p = 0.03–0.04).<br>67% in Group 1 and 23% in Group 2 showed penetration depth > 1.5 mm.<br>No statistical difference in RLL.	Adding a pressure injector to a cement gun showed a tendency of higher cement penetration to the posterior condyle of femur.
Lutz et al. (2009)	III	104 TKA patients<br><br>Group 1: 25<br>Group 2: 28<br>Group 3: 25<br>Group 4: 26	Group 1: Application with cement gun, performed by surgeon 1, between January 2000 and July 2001<br>Group 2: Application with finger packing, performed by surgeon 2, between January 2000 and July 2001<br>Group 3: Application with cement gun, performed by surgeon 1, between January 2002 and December 2003<br>Group 4: Application with cement syringe, performed by surgeon 2, between January 2002 and December 2003<br>Groups 1 and 3 had low-viscosity cement.<br>Groups 2 and 4 had standard-viscosity cement	Mean cement penetration depth in mm, (range) and number of RLL, respectively:<br>Group 1: 4.9 mm (4.4–5.4), 1 RLL<br>Group 2: 2.4 mm (2.2–2.7), 8 RLLs<br>Group 3: 5.0 mm (4.5–5.5), 2 RLLs<br>Group 4: 5.2 mm (4.7–5.7), 1 RLL<br><br>Usage of pressurized techniques (Groups 1, 2, and 3) had a significant effect on depth of cement penetration (p < 0.001) and reduction of RLL when compared with finger packing (p = 0.03).	There was no significant difference in RLL or cement penetration when comparing application by a cement gun with low-viscosity cement with a syringe with standard viscosity.<br><br>Cement penetration using finger packing was inferior compared with cement gun and syringe application.

Description	LoE	Material	Method of study	Outcome	Conclusion
Perez Mananes et al. (2012)	V	16 sawbones<br><br>Group 1: 4<br>Group 2: 4<br>Group 3: 4<br>Group 4: 4	Group 1: Finger packing after 2 min<br>Group 2: Finger packing after 5 min<br>Group 3: Cement directly applied as a handmade clay-model to the implant after 2 min<br>Group 4: Cement directly applied as a handmade clay-model to the implant after 5 min	Mean cement penetration depth in mm and (range):<br>Group 1: 4.5 (4.0–4.8) mm<br>Group 2: 3.0 (2.8–3.5) mm<br>Group 3: 2.8 (2.3–3.1) mm<br>Group 4: 2.1 (1.9–2.4) mm<br><br>Group 1 had significantly higher cement penetration than Group 4 (p = 0.007).<br>.	Finger packing the cement on the bone surface results in better cement penetration than applying it only to the implant after making a clay-model by hand.
Schlegel et al. (2014)	V	12 human cadaver tibiae<br><br>Group 1: 6<br>Group 2: 6	Group 1: pulsatile lavage and application with finger packing<br>Group 2: Syringe lavage and application with cement gun	Mean cement penetration, median cement layer thickness and median pull-out force, respectively:<br>Group 1: 1.5 mm, 1.2 mm, 8.8 kN<br>Group 2: 0.4 mm, 2.6 mm, 0.6 kN<br>The cement penetration was greater (p = 0.004) and interface strength was higher (p = 0.03) for Group 1.	Pulsatile lavage with finger packing improved cement penetration by a factor of 4 and interface strength by a factor of almost 12 when compared with syringe lavage and cement gun.
Schlegel et al. (2015a)	V	24 human cadaver tibiae<br><br>Group 1: 4<br>Group 2: 4<br>Group 3: 4<br><br>Control group: 12	One side of every pair was implanted by finger packing onto tibial surface (control group) and compared with their respectively treated side.<br>Group 1: Finger packing onto tibial surface and under tibial tray<br>Group 2: Finger packing onto tibial surface, under tibial tray and in stem channel<br>Group 3: Cement gun onto tibial surface	Cement penetration depth (SD) in treated group versus their control:<br>Group 1: 1.4 (0.5) mm vs. 1.6 (0.6) mm<br>Group 2: 1.1 (0.6) mm vs. 1.1 (0.2) mm<br>Group 3: 2.0 (1.0) mm vs. 1.1 (0.4) mm<br><br>No significant difference between the different groups and control (p = 0.07–0.9). Bone interface strength was similar between control and treatment side in all groups (p = 0.7). Group 3 had the highest mean penetration (2.0 mm) and showed a trend to be greater than the control side (p = 0.07).	Manual cement packing could be further warranted, as it has been shown to result in sufficient penetration and interface strength.<br><br>The group using a cement gun to apply cement had a trend towards the highest cement penetration.
Silverman et al. (2014)	V	10 human cadaver tibiae<br><br>Group 1: 5<br>Group 2: 5	Group 1: Application with cement gun in liquid phase<br>Group 2: Application with finger packing in a doughy phase	The mean cement penetration difference between Group 1 and Group 2:<br>Zone 1: 1.0 mm<br>Zone 2: 1.3 mm<br>Zone 3: 1.1 mm<br>Zone 4: 0.2 mm<br>The mean average cement penetration was greater for Group 2.<br>There was higher cement penetration in 3 of 4 zones (p = 0.03–0.05).	The use of cement in a doughy phase and not too early application of cement provided a deeper cement penetration into the proximal tibia.
Vanlommel et al. (2011)	V	25 sawbones<br><br>Group 1: 5<br>Group 2: 5<br>Group 3: 5<br>Group 4: 5<br>Group 5: 5	Group 1: 10 g cement applied directly on tibial component<br>Group 2: 20 g cement applied directly on tibial component<br>Group 3: 10 g applied on tibial component and 10 g on tibial bone by cement spatula<br>Group 4: 10 g applied on tibial component and 10 g on tibial bone by finger packing<br>Group 5: 20 g applied on tibial bone by cement gun	Cement penetration depth:<br>Group 1: 2.2 mm<br>Group 2: 2.6 mm<br>Group 3: 3.7 mm<br>Group 4: 3.8 mm<br>Group 5: 5.6 mm<br><br>No significant difference between Groups 1 and 2.<br>Group 5 had significantly higher cement penetration than the rest (p < 0.001).<br>Groups 3 and 4 were significantly better than Group 1 and 2 (p < 0.001) but no significant difference between the two.	Applying cement to the tibial baseplate and tibial bone leads to an optimal cement penetration of 3–5 mm.<br><br>Application by cement gun gives the best penetration depth but it could be excessive (Thermal damage theory, Huiskes et al. 1981).

LoE: level of evidence.
[a] TKA: total knee arthroplasty. [b] KSFS: Knee Society Function Score. [c] KSAS: Knee Society Assessment Score.
[d] ROM: range of motion. [e] RLL: radiolucent lines

Table 2. Surface versus full cementation

Description	LoE	Material	Method of study	Outcome	Conclusion
Arora and Ogden (2005)	IV	125 TKAs in 117 patients	Patients who underwent TKA with SC were assessed using postoperative radiography	There were 7 revisions because of aseptic loosening and 6 because of infections. Cumulative 10-year survival was 93% with aseptic loosening and osteolysis as end-point.<br>RLL was seen in 41 knees, 19 of these were above 2 mm and progressive.	The high rate of osteolysis and RLL could be because of wear from rotationally loose, press-fit patellar component or the use of surface cementation.
Bert and McShane (1998)	V	5 tibial models made from Ultem 1100 and Dario RF100	Group 1: SC [a] with 1 mm cement mantle<br>Group 2: FC [b] with 1 mm cement mantle<br>Group 3: SC with 1 mm cement mantle and cement finger packed into the tibial canal<br>Group 4: SC with 3 mm cement mantle<br>Group 5: FC with 3 mm cement mantle	Group 1 was the only group with increased micromotion when tested by liftoff. Groups 2, 3, and 4 were identical to group 5.<br><br>No statistics were reported.	Full cementation is superior with 1 mm cement mantle. Full and surface cementation were equal as long as the cement mantle was 3 mm.
Cawley et al. (2012)	V	13 knees, sawbones and use of finite element analysis to assess bone stress and strain	Group 1: FC<br>Group 2: SC<br>Group 3: Intact tibia (control)	Group 2 had a higher strain than Group 1 in 8 of 16 measurements ($p < 0.05$).<br>Bone remodeling simulations predicted more extensive bone resorption under the baseplate for full cementation (43%) than for surface cementation (29%).	SC gives a higher strain on the bone but lower bone resorption. This suggests that SC reduces the contribution of proximal bone resorption to aseptic loosening of the tibial component. The higher level of proximal stress shielding for FC is predicted to result in a larger area of bone resorption under the baseplate which could lead to aseptic loosening.
Chong et al. (2011)	V	5 knees made in computer model	Group 1: FC<br>Group 2: SC<br>Group 3: Cementless, no ingrowth to bone<br>Group 4: Cementless, partial ingrowth to bone<br>Group 5: Cementless, fully ingrowth to bone	Predicted bone resorption at tibial resected surface (at 60 months):<br>Group 1 and Group 5: 26–29%, Group 4: 17%, Group 2 and Group 3: 11%.<br>At 20 mm below resected surface: Bone resorption was observed for Group 1, Group 5, and Group 4 to a lower extent than at the resected surface.	The study suggested SC to induce less bone resorption. Greater bone resorption would inevitably weaken the bone supporting the tibial prosthesis, leading to potential problems such as aseptic loosening.
Galasso et al. (2013)	IV	150 TKAs selected from a group of 232 TKAs in patients<br><br>Group 1: 75<br>Group 2: 75	Group 1: SC<br>Group 2: FC	There was no significant difference between Group 1 and Group 2 when comparing ROM ($p = 0.1$), KSS [c] ($p = 0.7$), FTMA [d] ($p = 0.06$), or survival rate with revision as endpoint ($p = 0.9$).<br>Comparing survival rate with mechanical reason for revision gave Group 1 a better survival ($p = 0.03$).<br>No difference in overall survival between Group 1 and 2 ($p = 0.9$).	There was a higher survivorship for patients who underwent SC than FC when revision for any mechanical reasons were set as endpoint.<br>The mid-term analysis results in no difference between SC or FC in form of function.
Grupp et al. (2017)	V	24 human cadaver knees<br><br>Group 1: 6<br>Group 2: 6<br>Group 3: 6<br>Group 4: 6	Group 1: SC and 40 mm keel<br>Group 2: SC and 28 mm keel<br>Group 3: FC and 40 mm keel<br>Group 4: SC and 120 mm keel	No exact measurements of cement penetration. Mean load to failure (SD) (kN):<br>Group 1: 4.7 (1.1)<br>Group 2: 4.6 (1.4)<br>Group 3: 4.9 (0.7)<br>Group 4: 5.6 (0.5)<br>There was no statistical difference between the groups regarding mean load to failure ($p = 0.5–1.0$), cement penetration ($p = 0.7–0.9$), or stability.	This study showed no statistically difference when comparing SC, FC, or keel length

Description	LoE	Material	Method of study	Outcome	Conclusion
Hyldahl (2003)	II	80 TKAs in 77 patients<br><br>Group 1: 20<br>Group 2: 20<br>Group 3: 20<br>Group 4: 20	Group 1: FC with metal-backed component<br>Group 2: SC with metal-backed component<br>Group 3: FC with all-polyethylene component<br>Group 4: SC with all-polyethylene component	Group 1 and 2 comparison: No difference in clinical score, or postoperative radiographs.<br>Group 2 had higher rotation (p = 0.005) and liftoff (p = 0.04) and a tendency to a higher maximal point of motion.<br>Groups 3 and 4 were comparable: No difference in clinical score. No difference in radiostereometric analysis.	FC can be preferred when using a metal-backed tibial model. SC and FC were found equal when using an all-polyethylene model.
Hyldahl et al. (2005a)	II	36 TKAs.<br><br>Group 1: 20<br>Group 2: 16	Group 1: SC in all-polyethylene tibial components<br>Group 2: SC in metal-backed tibial components	The clinical assessment scores between the groups were not significantly different postoperatively at the 2-year follow-up (p = 0.7 and 0.4).<br>Metal-backed components migrated more (p-value 0.003–0.02) and had higher lift-off (p = 0.001 at 3 months, p > 0.1 at 12 and 24 months).	All-poylethylene components had a better fixation than metal-backed tibial components when using SC.
Hyldahl et al. (2005b)	II	40 TKAs in 39 patients<br><br>Group 1: 20<br>Group 2: 20	Group 1: FC in all-polyethylene prosthesis<br>Group 2: FC in metal-backed prosthesis	No difference in preoperative and postoperative assessment score at 2-year follow-up (p = 0.7 and p = 0.2).<br>There were no statistical differences between the groups with regard to rotations, translations, and migration at any time period. P-values not presented.	Metal-backed tibial components give no better fixation than all-polyethylene tibial components when the components are completely cemented.
Hofmann et al. (2006)	IV	107 TKAs in 88 patients	Retrospective review of patients with SC	ROM averaged from 1° to 116° postop.<br>KSS improved from mean 122 to 195.<br>No radiographs demonstrated osteolytic lesions. Non-progressive RLL were noted in 3 tibiae.<br>Mean depth of penetration was 2.7 mm.	SC allows reliable cement penetration and durable clinical results at 5 years' follow-up.
Luring et al. (2006)	V	10 sawbones<br><br>Group 1: 5<br>Group 2: 5	Group 1: FC<br>Group 2: SC	Comparing liftoff, in µm, with anterior, lateral, medial, and posterior loading.<br>Group 1: 10.6, 2.7, 3.7 and 7.7<br>Group 2: 28.5, 15.5, 18.6 and 17.4<br>Group 2 had a significantly higher maximum liftoff in all zones (p = 0.02–0.0001).	Cementing the stem reduced micromotion in mobile bearing knees.
Pelt et al. (2014)	IV	439 TKA patients	Retrospective review of patients with SC	RLLs that were found in Zone 1: 3 knees (< 1%), Zone 4: 7 (1.5%).<br>19 tibial components were removed (4%) within 5 years, 6 for aseptic loosening and 13 due to sepsis.	SC gives good survival in TKAs.
Peters et al. (2003)	V	24 human cadaver knees<br><br>Group 1: 8<br>Group 2: 8<br>Group 3: 8	Group 1: FC versus SC with a cruciated stem<br>Group 2: FC versus SC with an I-beam stem<br>Group 3: Cruciated versus I-beam both with SC	Group 1: No significant differences in micromotion after stress test.<br>Group 2: SC showed significantly less micromotion on the medial part.<br>Group 3: No significant differences.<br>All Groups: No difference in cement penetration. No p-values.	No difference between SC and FC in cement penetration.<br>SC showed less micromotion in the medial compartment in an I-beam prosthesis.
Rossi et al. (2010)	IV	70 TKAs in 62 patients	Patients who underwent SC under tray and at tibial bone surface were evaluated postoperatively	Mean KSS: 91. Mean function score: 86.<br>Cement penetration > 2 mm in all zones.<br>No early loosening was detected.<br>RLL was found in 7% of the patients.	Short-term follow-up gives good results for SC when applying cement under tray and at tibial surface.
Saari et al. (2009)	II	30 TKA patients<br>Group 1: 15<br>Group 2: 15	Group 1: FC<br>Group 2: SC	No significant difference between the 2 groups in terms of KSKS [e], KSFS (p > 0.2) or migration after 2 years using RSA [f] (p > 0.2).	No difference in migration using SC or FC technique in a metal-backed tibial component.

Description	LoE	Material	Method of study	Outcome	Conclusion
Schlegel et al. (2015a)	V	24 human cadavers<br><br>Group 1: 4<br>Group 2: 4<br>Group 3: 4<br>Group 4: 12	Group 1: SC (finger packing on both prosthesis and tibial surface)<br>Group 2: FC<br>Group 3: Gun cementation and SC<br>Group 4: SC with finger packing (control)	Cement penetration depth (SD) (mm) in treated group versus their control:<br>Group 1: 1.4 (0.5) vs. 1.6 (0.6)<br>Group 2: 1.1 (0.6) vs. 1.1 (0.2)<br>Group 3: 2.0 (1.0) vs. 1.1 (0.4)<br>No significant difference between the different groups and control (p = 0.07–0.9). Bone interface strength was similar between control and treatment side in all groups (p = 0.7). Group 3 had the highest mean penetration (2.0 mm) and showed a trend to be greater than the control side (p = 0.07).	No statistical difference between FC and SC.
Schlegel et al. (2015b)	III	77 TKA patients<br><br>Group 1: 25<br>Group 2: 42	Group 1: SC<br>Group 2: FC	Mean (SD) preoperative KSS, postoperative KSS and 10-year survival rate when aseptic loosening was set as endpoint:<br>Group 1: 21 (16), 85 (14) points and 100%.<br>Group 2: 29 (15), 70 (23) points and 93.3% (95% CI 80.5–100%) (p = 0.4).<br>No difference in KSS, but a higher improvement in Group 1 (p = 0.04).	No statistical difference between FC and SC.
Sharkey et al. (2002)	V	212 knees in 203 patients	A retrospective study of patients who underwent TKA	Of all revisions due to aseptic loosening, 21% of the prostheses were uncemented and 10.5 % had SC.<br>Early loosening was associated with SC, but no numbers for knees or patients were given.	SC implants were associated with early failure.
Skwara et al. (2009)	V	20 human cadavers<br><br>Group 1: 10<br>Group 2: 10	Group 1: SC<br>Group 2: FC	Maximum total point motion (mm), maximum liftoff (mm), rotation in X-axis (°) and micromotion of > 2 mm (set as failed) after 10,000 load cycles.<br>Group 1: 0.9 mm, 0.1 mm, 0.6° and micromotion > 2 mm in 2 cases.<br>Group 2: 2.6 mm, 0.0 mm, 1.3° and micromotion > 2 mm in 6 cases.<br>No significant difference in prosthesis migration, liftoff and rotation (p = 0.2–0.6).<br>A tendency to higher failure rate (micromotion > 2 mm) in group 2 (p = 0.07).	A tendency for higher failure rate in the FC group, but not statistically significant.

For abbreviations, see Table 1 and below.
[a] SC: surface cementation.
[b] FC: full cementation.
[c] KSS: Knee Society score.
[d] FTMA: femorotibial mechanical axis.
[e] KSKS: Knee Society Knee Score.
[f] RSA: Radiostereometric analysis

Table 3. Cement application area

Description	LoE	Material	Method of study	Outcome	Conclusion
Bauze et al. (2004)	V	20 porcine tibiae<br><br>Group 1: 5<br>Group 2: 5<br>Group 3: 5<br>Group 4: 5	Group 1: Finger packing on bone<br>Group 2: Undersurface of prosthesis coated with cement<br>Group 3: Finger packing on bone and fixation pressure by a novel clamp-cement pressurizer<br>Group 4: No implant (Control)	The % of cement penetration at 1, 2, 3, 4 and 5 mm, and overall stiffness (SD) (kN/mm)<br>Group 1: 55, 42, 14, 4, and 3. 1.4 (0.4)<br>Group 2: 78, 46, 21, 7, and 4. 2.0 (0.5)<br>Group 3: 64, 43, 15, 6, and 5. 1.5 (0.5)<br>Group 4: 0.9 (0.4) kN/mm<br>Cement penetration at 1 mm was greater for Group 2 compared with the rest ($p = 0.008$). There was no significant difference at deeper levels or between Group 3 and either of the 2 other groups at any level ($p > 0.3$ in all cases).<br>Significant differences in stiffness were found between different regions in the control group ($p < 0.001$), finger-packed cement group ($p = 0.02$), and undersurface cement group ($p = 0.04$).<br>Differences were not found after use of a cement pressurizer ($p = 0.6$).	Maximum penetration was achieved by coating the undersurface of the prosthesis and impaction of the cement with a mallet.<br>This method also produced a regional variation in stiffness which in turn causes regional variations in weaknesses.<br>Uniform stiffness was best achieved by the use of clamp pressurizer.
Grupp et al. (2013)	V	12 human cadaver knees in a UKA [a] model<br><br>Group 1: 6<br>Group 2: 6	Group 1: Application of cement under the tibial tray<br>Group 2: Application of cement onto tibial implant and on tibial bone	Mean (SD) cement thickness (mm), cement mantle (mm), load to failure (kN), and cement penetration (mm) in:<br>Group 1: 2.1 (0.3), 0.8 (0.4), 2.6 (0.7) and 1.5 (0.4)<br>Group 2: 2.3 (0.3), 1.0 (0.5), 2.8 (0.9) and 1.6 (0.4)<br>No significance differences in cement thickness ($p = 0.3$), cement mantle ($p = 0.5$), load to failure ($p = 0.7$), or cement penetration ($p = 0.7$).	This study showed no difference between applying cement at the tibial component only and applying cement on both tibial bone surface and component when comparing stability, failure load, and cement thickness and penetration in UKAs.
Han and Lee (2017)	III	734 TKAs in 486 patients<br><br>Group 1: 403<br>Group 2: 331	Group 1: Cement applied on distal, anterior cut surface and posterior part of femoral component without digital pressurization<br>Group 2: Cement applied on distal and anterior cut surface with finger packing and whole cement surface of the femoral component	Group 2 had less radiographic loosening ($p = 0.02$), less revision for aseptic loosening ($p = 0.03$) and a significant difference in survival when radiological femoral component loosening was set as endpoint in univariable analysis, HR 4.2, (1.3–14.2) ($p = 0.02$) and multivariable analysis, HR 3.1 (1.0–12.2) ($p = 0.048$).	Using finger packing and full cementation of the femoral component and bone gives less loosening.
Stannage et al. (2003)		46 TKA patients<br><br>Group 1: 20<br>Group 2: 11<br>Group 3: 15	Group 1: Cement applied under tray and stem<br>Group 2: Cement applied under tray and directly in tibial canal<br>Group 3: Cement applied under tray, directly in tibial canal, and use of negative suction	Group 1 had lower cement penetration in 4 of 5 zones compared with Group 2 and Group 3 ($p < 0.001$–0.05).<br>Group 2 had better cement penetration in 1 of 5 zones compared with Group 1 and Group 3 ($p < 0.05$).<br>Group 3 had better cement penetration in 4 of 5 zones compared with Group 1 and Group 2 ($p < 0.05$).<br>No exact measurements were stated, only given as figures.	Lavage and suction under the tibial baseplate achieved satisfactory penetration of cement.<br>Applying cement into the stem recess showed deeper penetration of cement on radiographs when compared with applying cement directly to the prosthesis keel.

Description	LoE	Material	Method of study	Outcome	Conclusion
Vanin-broukx et al. (2009)	V	20 sawbones<br><br>Group 1: 5 femurs<br>Group 2: 5 femurs<br>Group 3: 5 femurs<br>Group 4: 5 femurs	Group 1: 2/3 of the cement applied on bone; 1/3 of the cement applied on posterior component part<br>Group 2: All of the cement applied on bone<br>Group 3: All of the cement applied on the component<br>Group 4: cement divided equally between bone and component	Cement mantle (mm) in anterior and posterior cut, and cement penetration (mm) in anterior, distal, and posterior cut:<br>Group 1: 32, 17, 2.6, 3.7, and 3.4<br>Group 2: 32, 17, 2.7, 3.7, and 2.5<br>Group 3: 9.8, 9.1, 2.0, 3.7, and 2.0<br>Group 4: 24, 18, 3.0, 4,1, and 3.2<br><br>Group 1 was better than Group 3 and Group 4 comparing cement mantle thickness ($p < 0.007$).<br>Group 3 was inferior to the other groups in cement penetration in the central and posterior part of femur ($p < 0.05$).	Application of cement onto the anterior and distal bone surface, as well as the posterior part of femoral component (Group 1) gives a good cement penetration and cement mantle thickness.<br>Group 1 was superior to the other techniques.
Vanlommel et al. (2011)	V	25 sawbones<br><br>Group 1: 5<br>Group 2: 5<br>Group 3: 5<br>Group 4: 5<br>Group 5: 5	Group 1: 10 g cement applied directly on tibial component<br>Group 2: 20 g cement applied directly on tibial component<br>Group 3: 10 g applied on tibial component and 10 g on tibial bone by cement spatula<br>Group 4: 10 g applied on tibial component and 10 g on tibial bone by finger packing<br>Group 5: 20 g applied on tibial bone by cement gun	Cement penetration depth in:<br>Group 1: 2.2 mm<br>Group 2: 2.6 mm<br>Group 3: 3.7 mm<br>Group 4: 3.8 mm<br>Group 5: 5.6 mm<br><br>No significant difference between Groups 1 and 2.<br>Group 5 had significantly higher cement penetration than the rest ($p < 0.001$).<br>Groups 3 and 4 were significantly better than Group 1 and 2 ($p < 0.001$), but no significant difference between the two.	Applying cement to the tibial baseplate and tibial bone leads to an optimal cement penetration of 3 to 5 mm.<br><br>Application by cement gun gives the best penetration depth but it could be excessive (Thermal damage theory, Huiskes et al. 1981).
Wetzels et al. (2018)	V	5 pairs of human cadaver knees<br><br>Group 1: 5 tibiae<br>Group 2: 5 tibiae<br>Group 3: 5 femurs<br>Group 4: 5 femurs	Group 1: Cement applied onto the cut tibial bone<br>Group 2: Cement applied onto the cut tibial bone and tibial prosthesis<br>Group 3: Cement applied only to the femoral component<br>Group 4: Cement applied onto the anterior and distal part of femoral bone and posterior condyles of the femoral component	Cement penetration depth (SD) (mm) and cement distribution along the anterior surface in Group 3 and 4:<br>Group 1: 2.7 (0.2) mm<br>Group 2: 3.5 (0.5) mm<br>Group 3: 2.9 (0.4) mm and 1.0 mm<br>Group 4: 2.8 (0.4) mm and 2.5 mm<br><br>Group 2 was significantly better than Group 1 ($p = 0.007$).<br>No significant difference in cement penetration between Groups 3 and 4 but Group 4 had significantly better cement distribution on the anterior surface ($p = 0.01$).	Applying cement to both the tibial prosthesis and bone gave better cement penetration than applying cement only to the bone.<br>Applying cement to the posterior part of the femoral component and to the femoral bone gave better cement distribution.

For abbreviations, see Table 1
[a] UKA: Unicomparmental knee arthroplasty

Table 4. Bone irrigation

Description	LoE	Material	Method of study	Outcome	Conclusion
Boontana-pibul et al. (2016)	V	10 human cadaver bone<br><br>Group 1: 5<br>Group 2: 5	Group 1: Standard pulsatile lavage<br>Group 2: Pressurized CO_2 lavage and pulsatile lavage	Group 1: 1.2 mm cement penetration<br>Group 2: 1.9 mm cement penetration<br><br>Group 2 had significantly deeper cement penetration (p = 0.004).	The use of pressurized CO_2 combined with normal pulsatile lavage may give better cement penetration.
Clarius et al. (2009)	III	112 UKA [a] in 100 patients<br><br>Group 1: 56<br>Group 2: 56	Group 1: Pulsatile lavage<br>Group 2: Syringe lavage	Cement penetration in mm and RLL in %<br>Group 1: 2.6 mm. RLL 4%<br>Group 2: 1.5 mm. RLL 22%<br><br>Group 1 had a lower rate of RLL (p= 0.02) and higher average cement penetration (p < 0.0001).	Pulsatile lavage improved cement penetration and decreased the incidence of RLL.
Helwig et al. (2013)	V	12 human cadaver tibiae<br><br>Group 1: 3<br>Group 2: 3<br>Group 3: 3<br>Group 4: 3	Group 1: Pulsatile jet-lavage (Stryker)<br>Group 2: Fracture brush cleaning<br>Group 3: Manual irrigation with syringe<br>Group 4: No cleaning (control)	Cement penetration:<br>Group 1: 3.0 mm<br>Group 2: 1.8 mm<br>Group 3: 1.9 mm<br>Group 4: 1.3 mm<br><br>Cement contact distance and penetration was better in Group 1 compared with the rest (p < 0.001 and p < 0.001).	The result of this experimental study supports the use of pulsatile jet-lavage before cementing the tibial component in TKAs.
Jaeger et al. (2013)	V	20 human cadaver tibiae used for UKA<br><br>Group 1: 10<br>Group 2: 10	Group 1: Pulsatile lavage<br>Group 2: Syringe lavage	Mean maximum cement penetration:<br>Group 1: 5.8 mm<br>Group 2: 4.6 mm<br><br>Group 1 had a higher cement penetration in volume and depth (p < 0.001 and p < 0.001).	Pulsatile lavage should be considered as an adjunct to increase cement penetration in cemented UKA.
Maistrelli et al. (1995)	V	48 human cadaver tibial segments from 12 patients	Group 1: Pulsatile lavage for 1 min<br>Group 2: Syringe lavage for 1 min	Average cement penetration:<br>Group 1: 1.2 mm<br>Group 2: 0.3 mm	Pulsatile lavage results in higher cement penetration than syringe lavage.
Ritter et al. (1994)	III	363 TKAs in 221 patients<br><br>Group 1: 155<br>Group 2: 61<br>Group 3: 147	Group 1: Irrigation with a syringe and cement application by finger packing<br>Group 2: Lavage and cement application by finger packing<br>Group 3: Lavage and pressure injections of cement	Exact numbers of RLL are not stated.<br>There was a significantly higher amount of RLL in the femoral component in Group 1 compared with Groups 2 and 3 (p = 0.04–0.01).<br>The rate of RLL both around the entire tibial component and adjacent to the femoral component was greater in Group 1 than in Group 3 (p = 0.02 and p < 0.001, respectively).	Lavage of the osseous surface improves cement penetration and reduces occurrence of RLL.
Scheele et al. (2017)	V	18 human cadaver tibiae tested with UKA<br><br>Group 1: 6<br>Group 2: 6<br>Group 3: 6	Group 1: Pulsatile lavage<br>Group 2: Nylon cleaning brush<br>Group 3: No cleaning of the bone	Cement penetration depth, cement mantle thickness and cement depth below the resection line, respectively:<br>Group 1: 2.0 mm, 0.6 mm, 1.5 mm<br>Group 2: 2.1 mm, 0.4 mm, 1.8 mm<br>Group 3: 1.8 mm, 0.5 mm, 1.5 mm<br><br>Group 3 had a lower area with cement under the tibial component (p = 0.007).<br>The cement mantle was thicker in Group 1 than in Group 2 (p = 0.02).<br>No overall cement penetration depth differences between the groups.<br>No difference in compression test (p = 0.9).	Bone preparation significantly improves cement interdigitation.<br>The brush and pulsatile lavage showed similar cement penetration and stability.

Description	LoE	Material	Method of study	Outcome	Conclusion
Schlegel et al. (2011)	V	12 human cadaver tibiae<br><br>Group 1: 6<br>Group 2: 6	Group 1: Prepared by pulsatile lavage<br>Group 2: Irrigated by a bladder syringe	Cement penetration (mm) and mean pull-out force (kN), respectively:<br>Group 1: 1.3, 1.3<br>Group 2: 0.8, 0.6<br><br>Group 1 had higher cement penetration (p = 0.03) and higher mean pull-out force (p = 0.03).<br>Group 1 had all the failures at the cement–implant interface.<br>Group 2 had 5 out of 6 failures at the cement–bone interface.	Pulsatile lavage gives superior cement penetration and increased pull-out force compared with irrigation by syringe.
Schlegel et al. (2014)	V	12 human cadaver tibiae<br><br>Group 1: 6<br>Group 2: 6	Group 1: Pulsatile lavage and cement application with finger packing<br>Group 2: Syringe lavage and cement application with cement gun	Mean cement penetration, median cement layer thickness, and median pull-out force, respectively:<br>Group 1: 1.5 mm, 1.2 mm, 8.8 kN<br>Group 2: 0.4 mm, 2.6 mm, 0.6 kN<br><br>Group 1 had significantly higher cement penetration (p = 0.004) and interface strength (p = 0.03).	Pulsatile lavage gives superior cement penetration and interface strength.

For abbreviations, see Table 1
[a] UKA: Unicomparmental knee arthroplasty

Table 5. Drilling holes

Description	LoE	Material	Method of study	Outcome	Conclusion
Ahn et al. (2015)	II	399 TKAs in 244 patients<br><br>Group1: 290. Low bone sclerosis<br>Group 2: 109. High bone sclerosis	Group 1: 2.0 mm hole diameter<br>Group 2: 4.5 mm hole diameter<br><br>Both groups had a 4 mm depth and an approximately 5 mm interval between the holes	The maximal depths of cement penetration in mm (range) and cumulative incidence rate of RLL after 12 and 24 months (%):<br>Group 1: 2.2 (0.0–3.4), 19 and 20<br>Group 2: 4.8 (3.1–6.3), 3.6 and 5.5<br><br>Group 1 had significantly lower cement penetration ($p < 0.001$) and higher rate of RLL after 12/24 months ($p = 0.005/0.004$).	Using multiple drilling holes with a large diameter of 4.5 mm can improve the depth of cement penetration and reduce the occurrence of RLLs after TKAs.
Diaz-Borjon et al. (2004)	II	30 TKAs in 15 patients<br><br>Group 1: 15<br>Group 2: 15	Both groups had 10 3×15 mm holes made by a tibial punch<br>Group 1: A punch pressurizer method<br>Group 2: Manual cement packing (control)	The average density of cement intrusion in Group 1 was 1.3 (95% CI 1.2–1.5) times higher than the control group. There were no exact measurements of cement penetration.	The use of a pressurizing instrument provides a significant increase in cement-bone penetration.
Mann et al. (2012)	V	Group 1: 5 research dogs	3.2 mm drilling holes [a]	There was significantly more cement–bone interdigitation along the central keel of the component than under the tibial tray ($p = 0.002$).	Multiple drilling holes over the tibial surface should improve fixation and reduce interface micromotion.
Miskovsky et al. (1992)	V	21 human cadaver tibiae with UKA<br><br>Group 1: 7<br>Group 2: 7<br>Group 3: 7	Group 1: 2.4×10 mm holes<br>Group 2: Normal cut tibial surface<br>Group 3: Subchondral bone (control group)	The average (SD) micromotion in posterior and anterior position (µm) and posterior liftoff (µm) after 10.000 load cycles:<br>Group 1: 12 (4), 14 (8), and 18 (11)<br>Group 2: 61 (53), 43 (37), and 100 (72)<br>Group 3: 61 (50), 33 (16), and 84 (101)<br><br>Group 1 had significantly less micromotion in both zones ($p = 0.02–0.01$) and less posterior lift-off ($p < 0.005$) than Group 2. The same results compared with control ($p < 0.04$ to $p < 0.003$).<br>No significant differences between Group 2 and Group 3.	Drilling holes may give better fixation in UKA tibial component.
van de Groes et al. (2013)	V	80 cement–bone cylinders was created from 10 human cadaver femora<br><br>Group 1: 20<br>Group 2: 20<br>Group 3: 20<br>Group 4: 20<br><br>10 specimens each for shear and tensile testing	Group 1: Unprepared cortical bone<br>Group 2: Cortical bone roughened with a rasp<br>Group 3: Three 3.2 mm hole diameter and 3 mm depth holes drilled through the cortex<br>Group 4: Cancellous bone	The average (SD) tension strength (MPa) and shear strength (MPa):<br>Group 1: 0.1 (0.1) and 0.1 (0.2)<br>Group 2: 0.2 (0.2) and 1.1 (0.4)<br>Group 3: 1.2 (0.5) and 1.8 (1.0)<br>Group 4: 1.8 (0.8) and 3.9 (1.3)<br><br>Group 3 had a significantly higher tension strength than Groups 1 and 2 ($p = 0.001$ and 0.003) and significantly higher shear strength than Group 1 ($p = 0.003$).	Drilling holes gives higher tension strength in the bone–cement interface.

For abbreviations, see previous Tables.
[a] Method from Allen et al. (2009).

Table 6. Suction

Description	LoE	Material	Method of study	Outcome	Conclusion
Banwart et al. (2000)	V	12 human cadaver tibiae<br><br>Group 1: 6<br>Group 2: 6	Group 1: Positive pressure intrusion (PPI) (Cement gun)<br>Group 2: Negative pressure intrusion (NPI)	No exact measurements were stated, only given as figures.<br>No significant difference in maximum temperature measured, region-to-region cement mantle depth and cement intrusion depth. Cement intrusion depth was higher in NPI near the suction tip ($p < 0.07$).	The better cement–bone interface could occur because (i) NPI gives extra cleaning by removing fat and fluid; (ii) NPI gives a better cement intrusion. NPI was not statistically better than PPI.
Bucher et al. (2015)	V	20 sawbones, experimental model<br><br>Group 1: 5<br>Group 2: 5<br>Group 3: 5<br>Group 4: 5	Group 1: Tourniquet<br>Group 2: No tourniquet<br>Group 3: No tourniquet and cancellous suction<br>Group 4: No tourniquet, cancellous suction and cement gun	Cement penetration in cm^2 (SD) centrally and peripherally, respectively:<br>Group 1: 3.2 (0.4) and 2.1 (0.2)<br>Group 2: 2.7 (0.4) and 1.9 (0.2)<br>Group 3: 3.4 (0.3) and 2.4 (0.3)<br>Group 4: 4.3 (0.4) and 3.2 (0.2)<br>Group 2 had lower cement penetration than the rest ($p < 0.0001$–0.008).<br>Group 4 had significantly higher central and peripheral cement penetration compared with the rest ($p < 0.0001$).	The use of cement gun, no tourniquet, and suction gives good penetration during TKA.
Dinh et al. (2016)	V	24 human cadaver tibiae<br><br>Group 1: 6<br>Group 2: 6<br>Group 3: 6<br>Group 4: 6	Group 1: NPI and Palacos cement<br>Group 2: NPI and Simplex cement<br>Group 3: Finger packing and Palacos cement<br>Group 4: Finger packing and Simplex cement	When the cement mantle thickness was included, the total cement penetrations were (SD) (mm):<br>Group 1: 3.1 (0.4)<br>Group 2: 3.7 (0.3)<br>Group 3: 2.8 (0.6)<br>Group 4: 3.7 (0.5)<br>Group 1 had a higher cement penetration than Group 3 in the proximal zone ($p = 0.004$). No significant difference between Group 2 and Group 4 ($p = 0.1$).<br>No significant difference between cement penetration in Group 1 and 2 in area of interest ($p = 0.7$).<br>Significantly higher cement penetration in Group 4 than in Group 3.	NPI can improve cement penetration when using Palacos cement.
Matthews et al. (2009)	IV	41 TKA patients	Cement was applied onto the tibia surface by a 20 mL syringe. A mantle of cement was applied on top of the pressurized cement and NPI was turned on to pull the cement down. Same technique on femoral component	Mean cement penetration under tray was 9.3 mm.<br>Mean cement penetration around stem was 2.9 mm medially and 1.3 mm laterally.	The technique using a modified 20 mL syringe, tourniquet, and NPI gives an effective cement penetration over the entire surface of the tibial plateau.
Norton and Eyres (2000)	III	127 TKA patients<br><br>Group 1: 12<br>Group 2: 82<br>Group 3: 15<br>Group 4: 18	Group 1: No suction and no tourniquet<br>Group 2: No suction and tourniquet<br>Group 3: NPI and no tourniquet<br>Group 4: NPI and tourniquet	Mean (SD) cement penetration in all zones:<br>Group 1: 2.1 (0.8) mm<br>Group 2: 1.8 (0.7) mm<br>Group 3: 4.3 (1.4) mm<br>Group 4: 4.0 (1.1) mm<br>Group 3 and 4 had a significant higher cement penetration than Groups 1 and 2 ($p < 0.01$) in all zones. No significant difference between Groups 3 and 4	Use of NPI by cancellous bone suction improves cement penetration. This could weigh against not using a tourniquet.
Stannage et al. (2003)	III	46 TKA patients<br><br>Group 1: 20<br>Group 2: 11<br>Group 3: 15	Group 1: Cement applied under tray and stem<br>Group 2: Cement applied under tray and directly in tibial canal<br>Group 3: Cement applied under tray, directly in tibial canal, and use of NPI	Group 1 had lower cement penetration in 4 of 5 zones compared with Group 2 and Group 3 ($p < 0.001$–0.05).<br>Group 2 had better cement penetration in 1 of 5 zones compared with Group 1 and Group 3 ($p < 0.05$).<br>Group 3 had better cement penetration in 4 of 5 zones compared with Group 1 and Group 2 ($p < 0.05$).<br>No exact measurements were stated, only given as figures.	Applying cement into the stem recess showed deeper penetration of cement on radiographs when compared with applying cement directly to the prosthesis keel.

Table 7. Cement properties and timing of cementation

Description	LoE	Material	Method of study	Outcome	Conclusion
Dahabreh et al. (2015)	V	24 bovine bone specimens<br><br>Group 1: 6<br>Group 2: 6<br>Group 3: 6<br>Group 4: 6	Group 1: Applied CMW1 cement on bone after 2 minutes of curing<br>Group 2: Applied CMW1 cement on bone after 4 minutes of curing<br>Group 3: Applied SmartSet cement on bone after 2 minutes of curing<br>Group 4: Applied SmartSet cement on bone after 4 minutes of curing	The shear strength of the bone–cement interface (SD) (MPa):<br>Group 1: 2.8 (1.3)<br>Group 2: 1.4 (0.9)<br>Group 3: 2.9 (1.2)<br>Group 4: 3.0 (1.1)<br><br>Group 2 had significantly lower cement–bone interface strength ($p < 0.05$).<br>No significant difference between Groups 1, 3, and 4 ($p = 0.8–0.9$)<br>.	The study showed that the time of application of the cement varies for the different cement types. CMW1 cement should be applied at 2 min.
Miller et al. (2014)	V	14 human cadaver knees<br><br>Group 1a: 7<br>Group 1b: 5<br>Group 2: 2	Group 1a: TKAs from post-mortem patients with service > 10 years<br>Group 1b: TKAs from post-mortem patients with service < 10 years<br>Group 2: TKAs made on cadaver bone (Control) to make statistical models	The contact fraction in % (SD) and cement depth (mm) in:<br>Group 1a: 6.2 (1.5) and 0.4 (0.1)<br>Group 1b: 23 (14) and 1.1 (0.7)<br>Group 1a had lower contact fraction and less cement depth than Group 1b ($p = 0.003$ and 0.03).<br>The only prosthesis with RLLs had been in service for 18 years.	The study supports the surgical concept of obtaining sufficient initial cement interlock (approximately 3 mm) because of the cement interlock decay over time.
Park et al. (2001)	V	Group 1: 1<br>Group 2: 1<br>Group 3: 1<br>Group 4: 1<br>Group 5: 1<br><br>Tested cement–cement interface by pushing 2 cement molds together	A cement interface was created at different times<br>Group 1: No interface (Control)<br>Group 2: Combined after 1 min of curing<br>Group 3: Combined after 2 min of curing<br>Group 4: Combined after 4 min of curing<br>Group 5: Combined after 6 min of curing	Cement–cement interface bond strength (SD) MPa:<br>Group 1: 66 (2.5)<br>Group 2: 60 (6.8)<br>Group 3: 54 (7.6)<br>Group 4: 52 (8.9)<br>Group 5: 38 (4.1)<br>There was no significant difference in strength between Groups 2–4.<br>Group 2 was only 8% weaker than Group 1.<br>Group 5 is 42% weaker than Group 1 and had only 50% bonding using SEM [a] analysis.	This study shows that when using a cementing technique where 2 surfaces are mated together should happen between 1 min and 4 min after the cement mixing has started.
Silverman et al. (2014	V	10 human cadaver tibiae<br><br>Group 1: 5<br>Group 2: 5	Group 1: Application with cement gun on the bone in liquid phase<br>Group 2: Application with finger packing on the bone in a doughy phase	The mean cement penetration difference between Groups 1 and 2:<br>Zone 1: 1.0 mm<br>Zone 2: 1.3 mm<br>Zone 3: 1.1 mm<br>Zone 4: 0.2 mm<br><br>The mean average cement penetration was greater for Group 2.<br>There was higher cement penetration in 3 of 4 zones ($p = 0.03–0.05$)	The use of cement in a doughy phase and not too early application of cement to the bone provided deeper cement penetration into the proximal tibia.
Walker et al. (1984)	IV & V	Radiographic study: 45 TKA patients<br><br>Experimental study: 12 cadaver tibiae	Radiographic study: The study investigated the cement penetration in the knees that were in good condition 2 years after operation<br>Experimental study: Cement was applied at either 2, 4, or 6 min at the bone surface. They also applied pressure-sensitive film to look at force applied to the knee when using a leg-lifting fixation method. The bone sections were also fixed for tension testing	Radiographic study: Average cement penetration was 1.5–2.0 mm in the upper surface and 2.0–2.5 mm around the peg.<br><br>Experimental: Penetration depths varied between 0.5 and 4.0 mm.	Cement penetration in good functioning knees were between 1.5 and 3 mm 2 years after operation, but ideal cement penetration was suggested as 3-4 mm. To achieve this cement penetration the pore size, pressure and mixing time must be considered. This can be done by using a leg-lifting method about 4 min after initial cement mixing.

For abbreviations, see previous Tables.
[a] SEM: scanning electron microscope.

Table 8. Stabilization of the implant during the curing phasen

Description	LoE	Material	Method of study	Outcome	Conclusion
Bauze et al. (2004)	V	20 porcine tibiae<br><br>Group 1: 5<br>Group 2: 5<br>Group 3: 5<br>Group 4: 5<br>The implants were impacted with a mallet.	Group 1: Finger packing on bone and manual fixation pressure until cured<br>Group 2: Undersurface of prosthesis coated with cement and manual fixation pressure until cured<br>Group 3: Finger packing on bone and fixation pressure by a novel clamp-cement pressurizer<br>Group 4: No implant (Control)	The % of cement penetration at 1, 2, 3, 4 and 5 mm, and overall stiffness (SD) (kN/mm)<br>Group 1: 55, 42, 14, 4, and 3. 1.4 (0.4)<br>Group 2: 78, 46, 21, 7, and 4. 2.0 (0.5)<br>Group 3: 64, 43, 15, 6, and 5. 1.5 (0.5)<br>Group 4: 0.9 (0.4) kN/mm<br><br>Cement penetration at 1 mm was significantly greater for Group 2 compared with the rest ($p = 0.008$).<br>There was no significant difference at deeper levels or between Group 3 and either of the 2 other groups at any level ($p > 0.3$ in all cases).<br>Significant differences in stiffness were found between different regions in the control group ($p < 0.001$), finger-packed cement group ($p = 0.02$), and undersurface cement group ($p = 0.04$).<br>Differences were not found after use of a cement pressurizer ($p = 0.6$).	Maximum penetration was achieved by coating the undersurface of the tibia component and impaction of the component with a mallet. This method also produced a regional variation in stiffness, which in turn causes regional variations in weaknesses.<br>Uniform stiffness was best achieved by use of a clamp pressurizer.
Diaz-Borjon et al. (2004)	II	30 TKAs in 15 patients<br><br>Group 1: 15<br>Group 2: 15	Group 1: A punch pressurizer method<br>Group 2: Same method as Group 1 without punch pressurizer (control)	The average density of cement intrusion in the Group 1 was 1.3 (95% CI 1.2–1.5) times higher than the control group.<br>There were no exact measurements of cement penetration.	The use of a pressurizing instrument provides a significant increase in cement-bond penetration.
Guha et al. (2008)	III	50 TKAs in 36 patients<br><br>Group 1: 25<br>Group 2: 25	Group 1: Single-stage cementation which involved cement applied onto the tibial and femoral cut surface at the same time. Knee was held in extension during the curing phase.<br>Group 2: Two-stage cementation which involved cement applied for each component, separately	Group 1 had significantly lower total number of RLLs in anterior–posterior view ($p < 0.05$). No difference in lateral view.<br>Group 1 had significantly less wide RLLs (≥ 4 mm) than Group 2 ($p < 0.05$).	Single-stage cementing was superior to the two-stage technique in terms of avoiding RLL.
Jaeger et al. (2012)	V	24 human cadaver tibiae<br><br>Group 1: 8<br>Group 2: 8<br>Group 3: 8	Group 1: Femoral force application point (FFAP) at 0°<br>Group 2: FFAP at 45°<br>Group 3: FFAP at 90°	No significant difference between Group 1 and 2 ($p = 0.8$) but a significant difference between Groups 2 and 3 ($p < 0.001$) in terms of FFAP.<br><br>Mean cement penetration pressure at the measure points anterior and at the implant keel in Group 2 was not significantly different ($p = 0.2$), but it was at the implant keel versus the posterior measure point ($p = 0.04$) in Group 2.	A flexion angle of < 45° does not influence the position of the FFAP significantly.<br>With flexion of > 45°, the FFAP shifts backwards and may lead to higher cement penetration pressure in the posterior region and tilting of the tibial component.
Kanekasu et al. (1997)	III & V	Group 1: 10 in lab and 20 in patient study<br>Group 2: 10 in lab and 20 in patient study<br><br>20 tibial plastic models to the force test and 40 knees obtained from 25 patients	A prototype of the tibial baseplate clamper was made. It was first tried out on a plastic model to do a force test<br><br>Group 1: Clamp fixation on tibial component<br><br>Group 2: Manual pressure applied to a tibial baseplate clamper	Mean cement penetration (SD) (mm) in medial, lateral, anterior, and posterior zone:<br>Group 1: 2.5 (1.0), 3.9 (0.8), 4.6 (1.0), and 3.8 (1.0)<br>Group 2: 2.3 (0.9), 3.0 (0.9), 3.1 (0.6), and 3.3 (0.8)<br><br>No statistics or p-values were calculated.	The tibial baseplate clamper could be a useful device for the fixation during TKAs because of the large initial pressure force.<br>The tibial baseplate clamper also had a larger depth of cement penetration compared with the pusher method.

For abbreviations, see Table 1.
